# Chemotherapy is not necessary for early-stage serous and endometrioid ovarian cancer after undergoing comprehensive staging surgery

**DOI:** 10.1186/s13048-020-00694-9

**Published:** 2020-08-09

**Authors:** Shuqing Li, Zhiling Zhu

**Affiliations:** Department of Obstetrics and Gynecology, Obstetrics and Gynecology Hospital of Fudan University, 128 Shenyang Road, Shanghai, 200090 China

**Keywords:** Ovarian cancer, Serous, Endometrioid, Chemotherapy, Overall survival

## Abstract

In order to investigate whether adjuvant chemotherapy is essential for patients with early-stage serous and endometrioid epithelial ovarian cancer, the present study collected data from the US Surveillance, Epidemiology and End Results database between 2004 and 2015. All subjects underwent comprehensive staging surgery and were diagnosed as stages IA-IIA, grade 1–2. A total of 2644 patients were enrolled in the present study, among which 1589 patients received platinum-based chemotherapy. Comparisons of categorical data were performed via χ^2^ tests. Variables with *P* < 0.05 in univariate analyses were further analyzed using multiple logistic regression. Selection bias from the heterogeneity of demographic and clinical characteristics was avoided using propensity score matching. Cox proportional hazards models were applied to estimate hazard ratios (HRs) and 95% confidence intervals (CIs), investigating the association between variables and 5-year overall survival. After the propensity score matching, there was an equal number of patients with or without chemotherapy (*n* = 925). The results of the present study indicated that those aged ≥65 years were at an increased risk of ovarian cancer, and the age was associated with poor prognosis (HR, 1.486; CI, 1.208–1.827; *P* < 0.001). Endometrioid carcinoma was associated with improved 5-year overall survival compared with serous cystadenocarcinoma (HR, 0.697; CI, 0.584–0.833; *P* < 0.001). Chemotherapy could not prolong the 5-year overall survival of patients with early-stage serous and endometrioid ovarian cancer (HR, 1.092; CI, 0.954–1.249; *P* = 0.201). These results demonstrated that adjuvant chemotherapy was unnecessary for patients with early-stage serous and endometrioid ovarian cancer after they underwent comprehensive staging surgery.

## Introduction

Ovarian cancer is the leading cause of cancer-associated mortality among patients with gynecological malignancies worldwide. Data from the Surveillance, Epidemiology and End Results (SEER) database indicate that distant stage epithelial ovarian cancer (EOC) accounts for 59%, regional stage for 20%, and localized stage for 15% of cases. A great majority of patients with advanced epithelial ovarian cancer undergo surgery and receive platinum-based chemotherapy, which has been recommended by National Comprehensive Cancer Network (NCCN) guidelines [1–3]. However, whether adjuvant chemotherapy should be used for patients with early-stage ovarian cancer after they have received surgery remains controversial. The 5-year recurrence rate for early-stage ovarian cancer is currently 15–25% [4–6].

The paclitaxel/carboplatin regimen has formed the cornerstone of chemotherapy in epithelial ovarian cancer over the past two decades, and has achieved a successful clinical response [7, 8]. Nevertheless, both agents have considerable side effects, ranging from anticipated myelosuppression, alopecia and gastrointestinal symptoms, to occasional severe neurotoxicity [9, 10]. Notably, patients gradually develop chemoresistance with diminishing benefit from subsequent regimens [11–14]. Therefore, avoiding unnecessary chemotherapy will decrease the risk of drug resistance, increase the chance of secondary surgery and effectively prolong the 5-year survival time of patients with early-stage disease. This relieves the psychological pressure and economic burden, and significantly improves the quality of life of the patients. Taking these needs into account, there is an urgent requirement to improve the current understanding of the significance of chemotherapy for patients with early-stage disease, and to provide more current information for clinical practice.

Serous cystadenocarcinoma and endometrioid carcinoma, which are the two most frequent subtypes of epithelial ovarian cancer, were investigated in the present study [15]. The study cohort was designed to enroll patients with stage IA-IIA, grade 1–2 serous and endometrioid ovarian cancer. They all received comprehensive staging surgery and were actively followed-up. Patients were divided into chemotherapy and non-chemotherapy groups. The chemotherapy groups received paclitaxel/carboplatin regimen every 3 weeks for 3–6 cycles. The present study aimed to identify the necessity of adjuvant chemotherapy in early-stage serous and endometrioid ovarian cancer, which may provide a reference for gynecological oncologists.

## Methods

### Data source

The data in the present study were extracted from the US SEER database maintained by the National Cancer Institute. SEER*Stat software, version 8.3.5 was downloaded from the official website (https://seer.cancer.gov/). This program collects data from population-based cancer registries that currently cover ~ 28% of the US population.

### Patient eligibility criteria

**Fig. 1:**
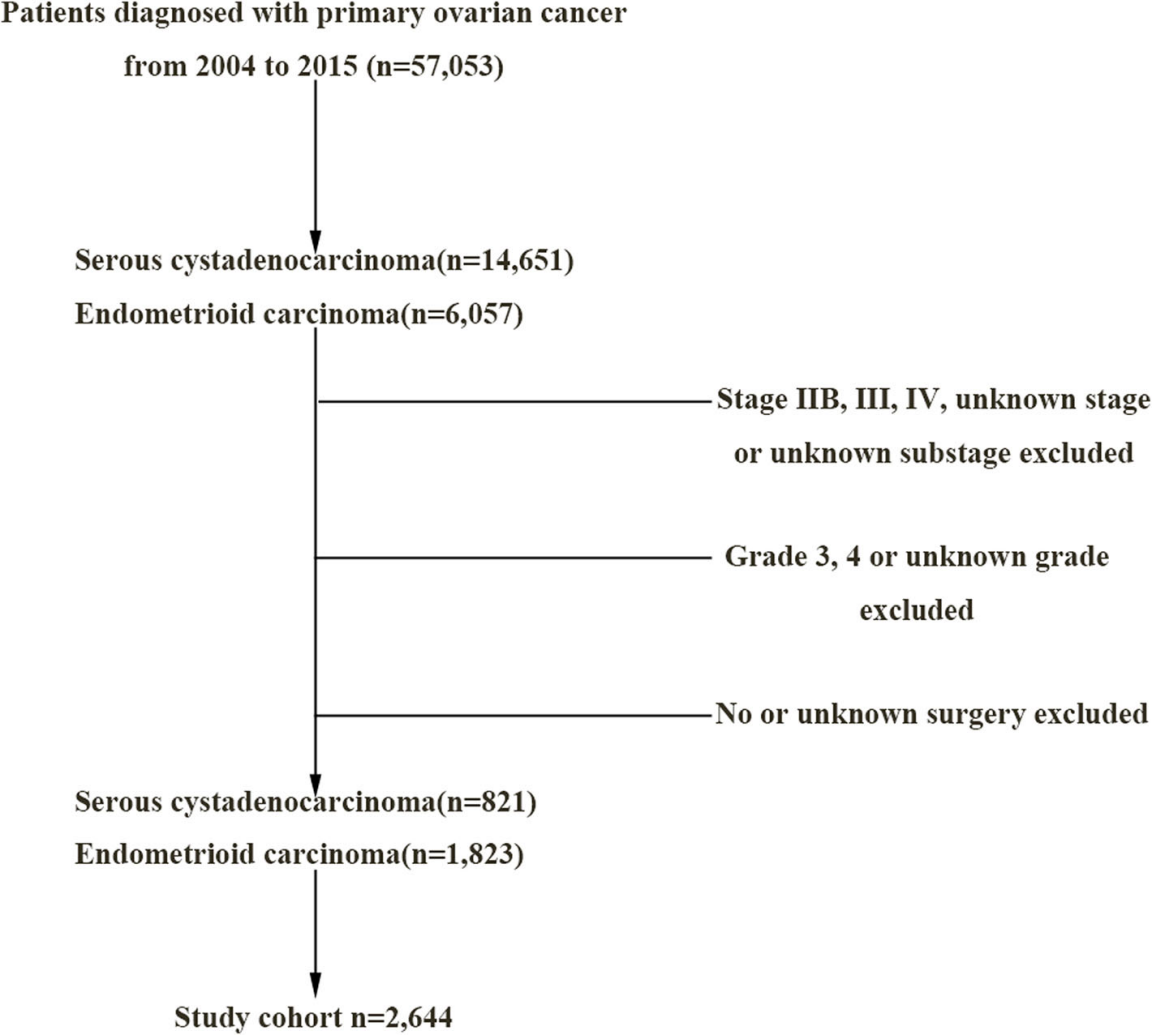
Consort diagram of patient selection

The study design and inclusion criteria are detailed in the aforementioned paragraph. A total of 2644 patients participated in the present study (Fig. 1). The following clinical characteristics were included: Patient ID, age at diagnosis, race, survival months, tumor stage, tumor grade, tumor laterality, tumor size, surgery of primary site, type of surgery, chemotherapy, vital status, cause-specific death classification, type of follow-up expected. Age at diagnosis was divided into three groups: < 45, 45–65 and > 65 years. Race was classified into white and non-white. Tumor laterality whereby the tumor originated was grouped into right, left and other/unknown. Tumor size was categorized into three groups: ≤10 cm, > 10 cm and unknown. Administration of chemotherapy fell into yes and no/unknown in the extracted dataset. The association between chemotherapy and 5-year overall survival (OS) was analyzed in all patients.

### Statistical analysis

#### Univariate analysis

There were no cases with missing data. Pearson χ^2^ tests were used to evaluate univariate associations between categorical variables and chemotherapy before and after the propensity score matching. All tests were two-sided. *P* < 0.05 was considered to indicate a statistically significant difference. Statistical analyses were performed using R software, version 3.5.1 and SPSS software, version 25.0.0.1.

#### Propensity score matching

Selection bias generally existed in retrospective studies due to the demographic heterogeneity and clinical characteristics between chemotherapy and non-chemotherapy groups. To lessen the influence of selection bias in the conclusions of the present study, propensity score matching was performed. A logistic regression model was applied to match age at diagnosis, race, tumor stage, tumor grade, tumor laterality, tumor size and histology between the two study groups. The propensity score ranged from 0 to 1. The present study adopted the nearest neighbor matching and 1:1 match ratio in this model.

#### Survival analysis

The survival analysis was performed using the Kaplan-Meier method, and differences were compared via log-rank tests. The present study performed cox proportional hazards models to estimate hazard ratios (HRs) and 95% confidence intervals (CIs).

## Results

### Patient demographics

The study inclusion criteria were met by 2644 eligible patients (1055 non-chemotherapy and 1589 chemotherapy). In this population, only 16.4% were aged < 45 years, 56.1% were aged 45–65 years, and 27.5% were aged ≥65 years. A large majority of patients were white (*n* = 2243; 84.8%). Stage IA accounted for 44.2%, stage IB for 5.7%, stage IC for 38.4%, and stage IIA for 11.7%. Of the 2644 patients, 59.1% were diagnosed with grade 1, and 40.9% with grade 2. Tumor laterality consisted of right (*n* = 1112; 42.1%), left (*n* = 1121; 42.4%) and other/unknown (*n* = 411; 15.5%). There were 44.3% of patients who had tumors < 10 cm in size, and 42.3% with tumors > 10 cm. In the present study, endometrioid carcinoma accounted for 68.9%, and serous cystadenocarcinoma for 31.1% of cases. Patients characteristics are presented in Table 1.

**Table 1:** Patient demographics

Characteristics	Number of patients	%
Chemotherapy		
No/Unknown	1055	39.9
Yes	1589	60.1
Age, years		
≤ 45	434	16.4
45–65	1484	56.1
> 65	726	27.5
Race		
White	2243	84.8
Non-white	401	15.2
Stage		
IA	1170	44.2
IB	152	5.7
IC	1014	38.4
IIA	308	11.7
Grade		
1	1562	59.1
2	1082	40.9
Laterality		
Right	1112	42.1
Left	1121	42.4
Other/Unknown	411	15.5
Tumor size, cm		
≤ 10	1171	44.3
> 10	1119	42.3
Unknown	354	13.4
Histology		
Serous	821	31.1
Endometrioid	1823	68.9

### Comparison of univariate covariates

Before the propensity score matching, patients in the chemotherapy groups tended to be younger than those in the non-chemotherapy groups (≤45 years: 17.2 vs. 15.3%; 45–65 years: 59.5 vs. 51.0%; *P* < 0.001). They were less likely to be in stage IA (34.2 vs. 59.3%; *P* < 0.001), and more likely to be in stage IB (5.8 vs. 5.7%; *P* < 0.001) and stage IC (46.0 vs. 26.8%; *P* < 0.001). Compared with non-chemotherapy groups, grade 1 cases were less among the chemotherapy groups (52.9 vs. 68.3%; *P* < 0.001) and there were more grade 2 cases (47.1 vs. 31.7%; *P* < 0.001). There was a larger number of patients that had serous cystadenocarcinoma in the chemotherapy groups (33.5 vs. 27.4%; *P* = 0.001). To eliminate the heterogeneity and imbalance between groups, the present study performed propensity score matching and a logistic regression analysis. The results demonstrated that the two groups both had an equal number of patients, and all clinical factors were well balanced without significant differences, indicating the potential covariates between groups were greatly decreased (Table 2).

**Table 2:** Comparison of univariate covariates

		Before PSM	*P*-value		After PSM	P-value
Characteristics	Chemotherapy- (*n* = 1055)	Chemotherapy+ (*n* = 1589)		Chemotherapy- (*n* = 925)	Chemotherapy+ (*n* = 925)	
Age			< 0.001			0.254
≤ 45	161 (15.3)	273 (17.2)		159 (17.2)	183 (19.8)	
45–65	538 (51.0)	946 (59.5)		504 (54.5)	503 (54.4)	
> 65	356 (33.7)	370 (23.3)		262 (28.3)	239 (25.8)	
Race			0.086			0.124
White	911 (86.4)	1332 (83.8)		793 (85.7)	768 (83.0)	
Non-white	144 (13.6)	257 (16.2)		132 (14.3)	157 (17.0)	
Stage			< 0.001			0.328
IA	626 (59.3)	544 (34.2)		499 (53.9)	475 (51.4)	
IB	60 (5.7)	92 (5.8)		57 (6.2)	49 (5.3)	
IC	283 (26.8)	731 (46.0)		283 (30.6)	319 (34.5)	
IIA	86 (8.2)	222 (14.0)		86 (9.3)	82 (8.9)	
Grade			< 0.001			0.530
1	721 (68.3)	841 (52.9)		595 (64.3)	581 (62.8)	
2	334 (31.7)	748 (47.1)		330 (35.7)	344 (37.2)	
Laterality			< 0.001			0.147
Right	477 (45.2)	635 (40.0)		407 (44.0)	383 (41.4)	
Left	461 (43.7)	660 (41.5)		405 (43.8)	401 (43.4)	
Other/Unknown	117 (11.1)	294 (18.5)		113 (12.2)	141 (15.2)	
Tumor size, cm			0.044			0.888
≤ 10	464 (44.0)	707 (44.5)		414 (44.8)	416 (45.0)	
> 10	429 (40.7)	690 (43.4)		386 (41.7)	391 (42.3)	
Unknown	162 (15.4)	192 (12.1)		125 (13.5)	118 (12.8)	
Histology			0.001			0.226
Serous	289 (27.4)	532 (33.5)		257 (27.8)	233 (25.2)	
Endometrioid	766 (72.6)	1057 (66.5)		668 (72.2)	692 (74.8)	

Data are expressed as n (%). *P* < 0.05 was considered to indicate a statistically significant difference. *PSM* propensity score matching

### Association between chemotherapy and survival

**Fig. 2:**
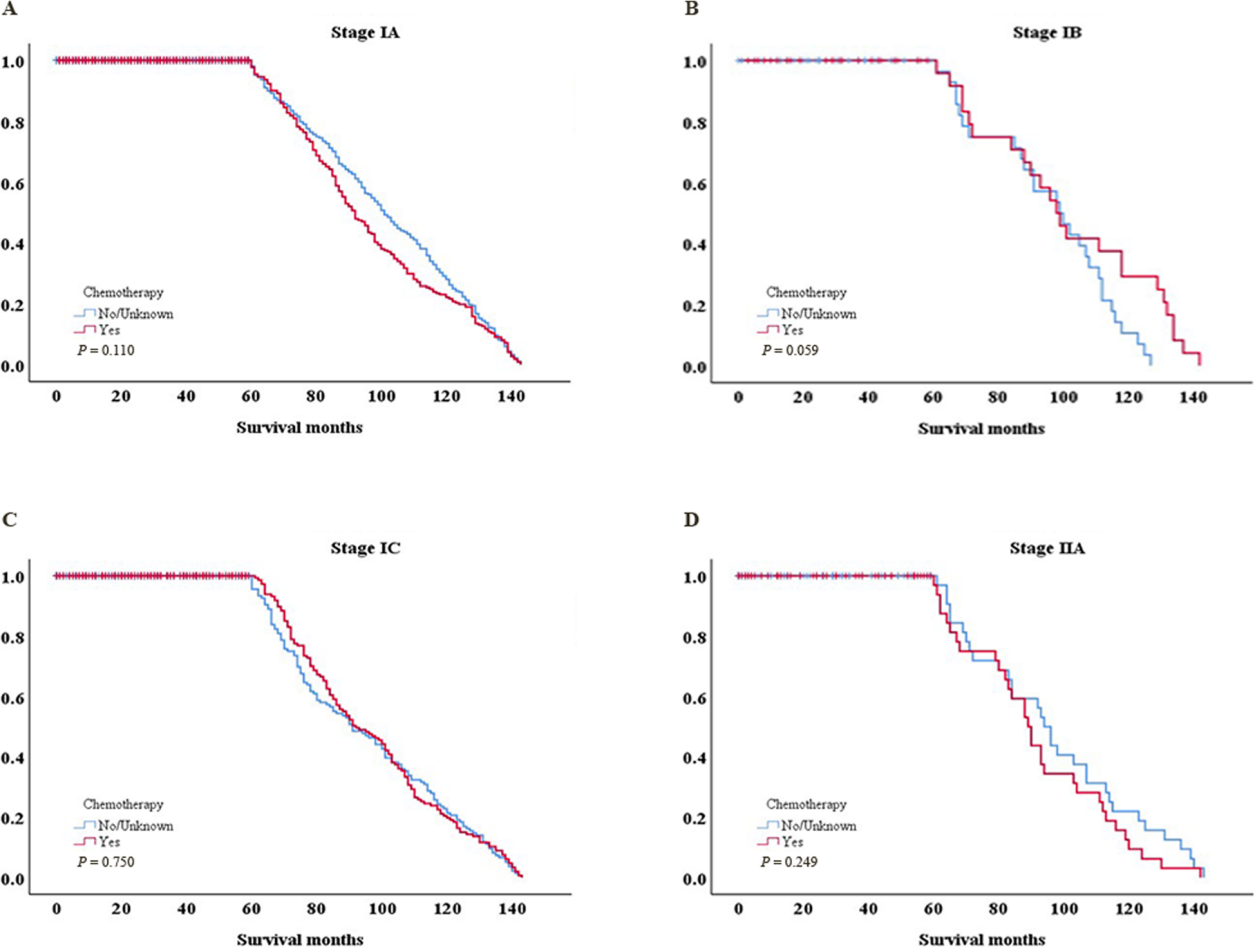
Kaplan-Meier survival curves for (**a**) stage IA, (**b**) stage IB, (**c**) stage IC and (**d**) stage IIA drawing on the basis of Table 3. *P* < 0.05 was considered to indicate a statistically significant difference

The present study analyzed the association between chemotherapy and 5-year OS for stages IA-IIA. There were no statistically significant differences between the chemotherapy and non-chemotherapy groups (stage IA: 46.5 vs. 53.7%; *P* = 0.110; stage IB: 49.0 vs. 49.1%; *P* = 0.059; stage IC: 46.1 vs. 48.1%; *P* = 0.750; stage IIA: 39.0 vs. 37.2%; *P* = 0.249). Patients with early-stage disease could not benefit from chemotherapy to prolong their 5-year OS (Table 3 and Fig. 2).

**Table 3:** Association of chemotherapy with 5-year overall survival

Stage	Chemotherapy- (*n* = 925)	Chemotherapy+ (*n* = 925)	*P*-value
IA	53.7%	46.5%	0.110
IB	49.1%	49.0%	0.059
IC	48.1%	46.1%	0.750
IIA	37.2%	39.0%	0.249

*P* < 0.05 was considered to indicate a statistically significant difference

### Univariate analysis of clinical factors with survival

**Fig. 3:**
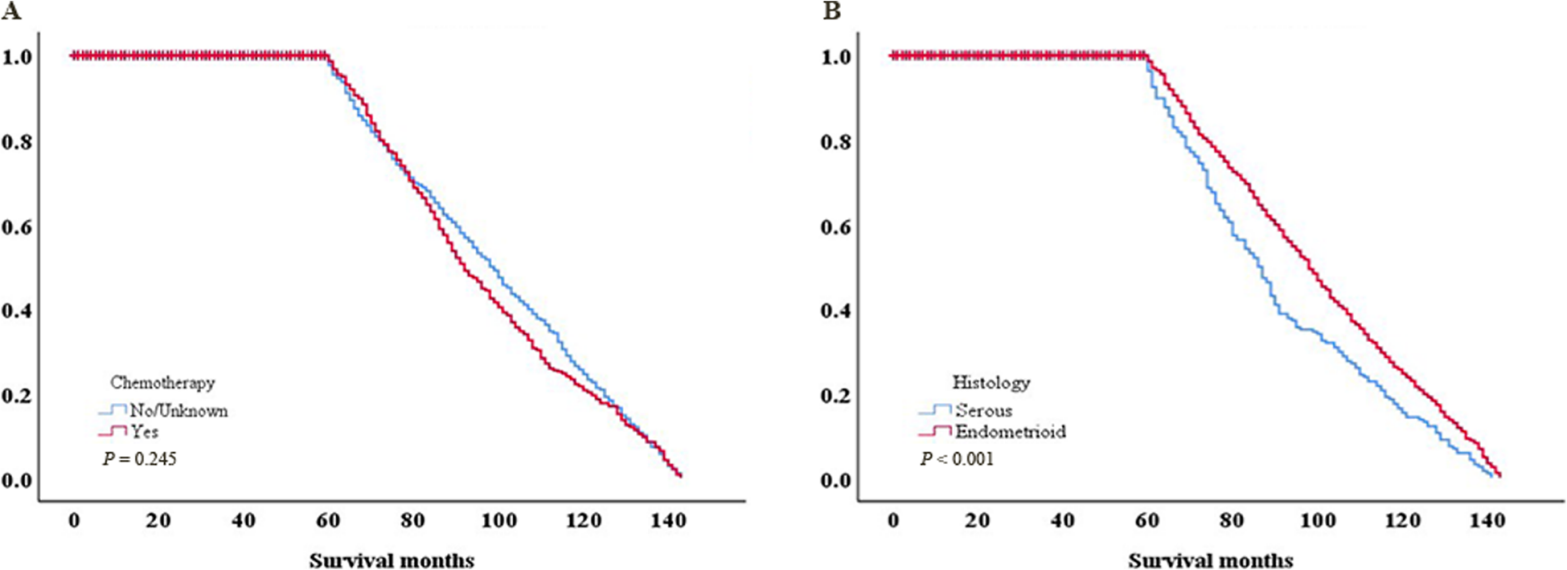
Kaplan-Meier survival curves for (**a**) chemotherapy and (**b**) histology drawing on the basis of Table 4. *P* < 0.05 was considered to indicate a statistically significant difference

The present study performed a univariate analysis of the matched population in order to investigate the prognostic effects of the clinical factors. No significant differences were observed between the chemotherapy and non-chemotherapy groups for 5-year OS (*P* = 0.245). Older age was a risk factor for 5-year OS (*P* < 0.001). Tumors > 10 cm in size had lower 5-year OS (*P* = 0.014). Furthermore, 5-year OS of endometrioid carcinoma was higher than serous cystadenocarcinoma (*P* < 0.001; Table 4 and Fig. 3).

**Table 4:** Univariate analysis of clinical factors with 5-year OS

Characteristics	No.	5-year OS, %	*P*-value
Chemotherapy			0.245
No/Unknown	925	50.2	
Yes	925	45.8	
Age, year			< 0.001
≤ 45	342	51.5	
45–65	1007	49.3	
> 65	501	43.1	
Race			0.833
White	1561	48.9	
Non-white	289	43.3	
Stage			0.354
IA	974	50.2	
IB	106	49.1	
IC	602	47.0	
IIA	168	38.1	
Grade			0.908
1	1176	51.2	
2	674	42.4	
Laterality			0.163
Right	790	46.7	
Left	806	50.5	
Other/Unknown	254	44.1	
Tumor size, cm			0.014
≤ 10	830	45.5	
> 10	777	44.8	
Unknown	243	66.7	
Histology			< 0.001
Serous	490	38.4	
Endometrioid	1360	51.5	

*P* < 0.05 was considered to indicate a statistically significant difference. *OS* overall survival

### Cox proportional hazards model

The cox proportional hazards model is often used in medical research to investigate the association between survival time of patients and one or more predictive variables. The Kaplan-Meier method and log-rank tests describe survival according to one factor under investigation, but to not include the impact of any others. In addition, they are available only when the predictive variables are categorical, and do not easily work for quantitative predictors, such as age. Given these issues, an alternative method is the cox proportional hazards regression analysis, which works for both quantitative predictive variables and categorical variables. Furthermore, the cox regression model extends survival analysis methods to simultaneously assess the effects of several risk factors on survival time.

In order to investigate how clinical factors jointly impact on survival, the present study took all the factors associated with survival into a multivariate cox regression analysis. The results are presented in Table 5. The analysis revealed that elderly patients (age, ≥65 years) had a higher risk and worse prognosis (HR, 1.486; CI, 1.208–1.827; *P* < 0.001). Endometrioid carcinoma was associated with improved 5-year OS (HR, 0.697; CI, 0.584–0.833; *P* < 0.001). Chemotherapy still had no statistically significant effect on the 5-year OS after excluding the influence of all the confounding factors (HR, 1.092; CI, 0.954–1.249; *P* = 0.201).

**Table 5:** Multivariate cox regression analysis for 5-year overall

Characteristics	HR (95% CI)	*P*-value
Chemotherapy		
No/Unknown	Ref	
Yes	1.092(0.954–1.249)	0.201
Age, years		
≤ 45	Ref	
45–65	1.044(0.877–1.244)	0.627
> 65	1.486 (1.208–1.827)	< 0.001
Race		
White	Ref	
Non-white	0.984 (0.810–1.195)	0.872
Stage		
IA	Ref	
IB	0.967 (0.689–1.359)	0.848
IC	1.063 (0.910–1.241)	0.441
IIA	1.128 (0.862–1.475)	0.381
Grade		
1	Ref	
2	0.865 (0.743–1.008)	0.063
Laterality		
Right	Ref	
Left	0.984 (0.852–1.136)	0.824
Other/Unknown	1.115 (0.862–1.443)	0.407
Tumor size, cm		
≤ 10	Ref	
> 10	0.984 (0.848–1.141)	0.830
Unknown	0.760 (0.630–0.918)	0.004
Histology		
Serous	Ref	
Endometrioid	0.697 (0.584–0.833)	< 0.001

*P* < 0.05 was considered to indicate a statistically significant difference. *HR* hazard ratios, *CI* confidence intervals, *Ref* reference

## Discussion

The present study was based on a large and unique population-based cohort. The large size of the present study provided the statistical power to investigate the necessity of adjuvant chemotherapy for early-stage serous and endometrioid ovarian cancer. The unique feature of the present study was that each patient included had previously undergone comprehensive staging surgery, which is paramount for survival [16–18]. However, several limitations should be noted, which were inherent to all SEER database analyses. Not all details of the primary surgery could be acquired. The importance of residual disease as a significant prognostic factor for outcome was clearly understood, but accurate surgical data were difficult to obtain for the majority of patients [7, 19]. Furthermore, the dataset lacked information concerning recurrence free survival and further treatment history affecting prognosis. Therefore, treatment groups may have exhibited additional high-risk features that the authors of the present study were not aware of. To decrease selection bias, propensity score matching was conducted to randomize the dataset and to strengthen causal arguments. Cox proportional-hazards models were recommended by the NCCN guidelines to analyze the associations between variables and survival. Finally, the results from the present study supported recent publications that questioned the value of adjuvant chemotherapy in early-stage epithelial ovarian carcinoma [20–22]. Adjuvant chemotherapy was not necessary for patients with early-stage serous and endometrioid epithelial ovarian cancer after undergoing surgery. An alternative to paclitaxel plus carboplatin has not been identified over the past two decades as the first-line primary chemotherapy for epithelial ovarian cancer [7]. The two canonical drugs decrease the rate of recurrence and mortality, but does not affect long term survival and cannot decrease the eventual likelihood of death from ovarian cancer per se [8]. This is the reasoning for continuing to put patients with early-stage disease through the regimens.

In summary, the present study suggested that patients with early-stage serous and endometrioid ovarian cancer had no need to receive adjuvant chemotherapy when comprehensive staging surgery had been performed. Further investigation is warranted to provide guidance in the management of patients with epithelial ovarian cancer. Evaluation by a gynecological oncologist is strongly recommended for all patients with suspected ovarian cancer. Primary assessment and surgery by a gynecological oncologist can incur a survival advantage. In addition, NCCN suggests that the best form of management for any patient with cancer is in a clinical trial. Thus, clinical trials are urgently required in order to identify patients who may benefit most from adjuvant chemotherapy and to identify the optimal therapeutic strategy.

## Data Availability

The datasets analyzed during the present study are available from the corresponding author on reasonable request.
